# Evaluation of Blood Coagulation Parameters and ADMA, NO, IL-6, and IL-18 Serum Levels in Patients with Neovascular AMD before, during, and after the Initial Loading Phase of Intravitreal Aflibercept

**DOI:** 10.3390/life11050441

**Published:** 2021-05-14

**Authors:** Michał Wiciński, Małgorzata Seredyka-Burduk, Sławomir Liberski, Daria Marczak, Magdalena Pol, Bartosz Malinowski, Katarzyna Pawlak-Osińska, Bartlomiej J. Kaluzny

**Affiliations:** 1 Department of Pharmacology and Therapeutics, Faculty of Medicine, Collegium Medicum in Bydgoszcz, Nicolaus Copernicus University, M. Curie 9, 85-090 Bydgoszcz, Poland; wicinski4@wp.pl (M.W.); dariamarczak1@wp.pl (D.M.); bartosz.malinowski@cm.umk.pl (B.M.); 2 Division of Ophthalmology and Optometry, Department of Ophthalmology, Faculty of Medicine, Collegium Medicum in Bydgoszcz, Nicolaus Copernicus University, M. Curie 9, 85-090 Bydgoszcz, Poland; mburduk@wp.pl (M.S.-B.); magdalena.pol@cm.umk.pl (M.P.); b.kaluzny@cm.umk.pl (B.J.K.); 3 Division of Human Anatomy and Physiology, Institute of Health Sciences, Pomeranian University of Słupsk, K. Arciszewskiego 22A, 76-200 Słupsk, Poland; osinskak1@wp.pl

**Keywords:** aflibercept, age-related macular degeneration, anti-VEGF, asymmetric dimethylarginine, coagulation parameters, interleukin 6, interleukin 18, intravitreal treatment, nitric oxide

## Abstract

We evaluated the effect of intravitreal injections of aflibercept (IVA) on blood coagulation parameters including prothrombin time (PT), activated partial thromboplastin time (APTT), and thrombin time (TT), as well as asymmetric dimethylarginine (ADMA), nitric oxide (NO), interleukin 6 (IL-6), and interleukin 18 (IL-18) serum levels in patients with neovascular AMD (nAMD). Twenty-two eyes of 22 patients with nAMD were included. Parameters were evaluated before and 2–3 days after the first IVA injection, and then immediately before and 2–3 days after the third IVA injection. We revealed prolongation of the TT after the initial loading phase of IVA (*p* = 0.041) and a significant increase in IL-18 serum concentration immediately before the third IVA administration compared to baseline (*p* = 0.037). There were no statistically significant differences of other parameters and PT, APTT, ADMA, NO, and IL-6 values remained within the normal range at each of the time points of the study. Our results suggest that repeated IVA administration may affect the common blood coagulation pathway, which manifests as a prolongation of the TT value. Furthermore, we showed a significant increase in serum concentration of the pro-inflammatory cytokineIL-18during the initial loading phase of IVA.

## 1. Introduction

The main mechanism leading to impaired central vision in patients with neovascular age-related macular degeneration (nAMD) is an abnormal growth of pathological blood vessels (neovascularization) within the central retina, which is mainly stimulated by a vascular endothelial growth factor (VEGF). This protein molecule plays a key role both in physiological as well as pathological angiogenesis in different tissues [1]. Additionally, VEGF has a protective effect on vascular endothelial cells preventing their apoptosis, and may also increase the permeability of microvessels [2]. Therefore, the introduction of intravitreally administered VEGF inhibitors was a milestone in the treatment of patients with nAMD, leading to a significant improvement in prognosis by significantly reducing the loss of vision in patients affected by this disease. Pegaptanib, the first anti-VEGF agent, was introduced in the mid-2000s, but later anti-VEGF agents—ranibizumab, bevacizumab, and aflibercept—were better tolerated and more effective [3].

Current research has shown that one of the main factors responsible for the pathogenesis of nAMD, along with local ischemia, is the presence of a chronic inflammatory process, both systemic and local, especially in retinal and choroid tissue. Inflammatory factors may interfere with the balance between pro and anti-angiogenic factors and promote choroidal neovascularization (CNV) [4]. Moreover, the inflammatory process may cause vascular endothelial dysfunction due to the induction of oxidative stress and an increase in the level of asymmetric dimethylarginine (ADMA) in the serum and lead to a decrease in the bioavailability of nitric oxide (NO), the key factor for the proper function of the vascular endothelium [5,6].

Aflibercept has been approved for the treatment of nAMD in the US and Europe, in 2011 and 2012, respectively [1]. In addition to nAMD, the Food and Drug Administration (FDA) also approved aflibercept for the treatment of neovascularization in pathological myopia, occlusion of the retinal vein, and macular edema in the course of diabetes [6]. In 2015, there were nearly 220,000 intravitreal injections in the USA, of which nearly 60,000 were aflibercept compared to 180,000 and 36,600 in 2014 [7]. Thus, it can be assumed that the increasing use of anti-VEGF drugs may lead to an increase in the number of adverse effects in the treated population. It has been proven that the systemic use of anti-VEGF agents in oncological practice is associated with an increased risk of cardiovascular events including stroke, congestive heart failure, and myocardial infarction [2]. Prospective clinical trials VIEW 1 and VIEW 2 investigating the systemic safety of intravitreal injections of aflibercept (IVA) showed that the intravitreal use of aflibercept (IVA) and ranibizumab (IVR) in the nAMD patients were characterized by comparable safety to control group; similar conclusions were obtained in the comparison of the safety profile of IVA, IVR, and intravitreal bevacizumab (IVB) in patients with diabetic macular edema (DME) [8].

Inhibition of VEGF may act synergistically with proinflammatory factors and decreased bioavailability of NO and lead to activation of the coagulation cascade. Moreover, it has been demonstrated that patients with nAMD have a greater risk of cardiovascular events compared to healthy individuals [9]; hence, the influence of anti-VEGF agents on systemic parameters that may reflect the risk of cardiovascular events, in particular, blood coagulation parameters in this group should be carefully verified. Our prospective, interventional study was designed to evaluate the effect of IVA on blood coagulation parameters including thrombin time (TT), prothrombin time (PT), activated partial thromboplastin time (APTT), and the proinflammatory cytokines—interleukin 6 (IL-6) and interleukin 18 (IL-18), as well as parameters assessing endothelial function—ADMA and NO serum levels of nAMD patients at the baseline, during, and after the initial aflibercept loading phase.

## 2. Materials and Methods

### 2.1. Patients

This prospective, pre-post interventional study including 22 eyes of 22 treatment-naive patients with previously diagnosed nAMD who received an initial loading phase of intravitreal injections of 2 mg/0.05 mL of aflibercept (Eylea, Bayer, HealthCare, Berlin, Germany) at the Department of Ophthalmology, Collegium Medicum NCU, Bydgoszcz, Poland. For all participants of the study, the baseline C-reactive protein (CRP) level was determined to eliminate the influence of inflammation on the obtained results. Exclusion criteria included cardiovascular disease, chronic kidney or liver disease, uncontrolled hypertension, use of anti-inflammatory drugs (e.g., steroids, NSAIDs) or anticoagulants (e.g., heparin, vitamin K antagonists, or direct thrombin inhibitors), and the presence of other blood clotting disorders (e.g., von Willebrand disease), as well as nicotinism and alcoholism. All participants of the study were informed verbally and in writing about the principles of the conducted examination, which was then confirmed by a written signature of consent to participate in the experiment prior to registration of participation. The study was designed and carried out in accordance with the Helsinki Declaration and after obtaining a positive opinion from the Local Bioethics Commission. Table 1 presents the clinical and demographic characteristics of the cohort included in the study.

### 2.2. Study Design 

All 22 patients received three monthly IVA injections of 2 mg/0.05 mL according to the recommended intravitreal aflibercept frequency regimen in the initial loading phase for patients with nAMD. All participants were subjected to a thorough ophthalmologic examination before inclusion in the study, which involved the assessment of visual acuity using Snellen Eye Test Charts, evaluation of the anterior segment of the eye, intraocular pressure (IOP) measurement, and examination of the posterior segment with spectral-domain optical coherence tomography (SD-OCT), as well as fluorescein angiography (FA). Furthermore, the medical history and other relevant clinical data ofqualified participants were collected.

**Figure 1:**
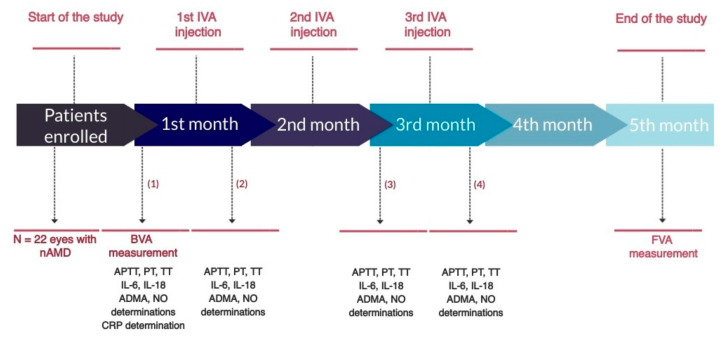
Timeline for the study. Measurement collected: (1) immediately before the first IVA injection; (2) 2–3 days after the first IVA injection; (3) immediately before the third IVA injection; (4) 2–3 days after the third IVA injection. Abbreviations: ADMA, asymmetric dimethylarginine; APTT, activated partial thromboplastin time; BVA, baseline visual acuity; FVA, final visual acuity; IL-6, interleukin 6; IL-18, interleukin 18; IVA, intravitreal aflibercept; NO, nitric oxide; PT, prothrombin time; TT, thrombin time.

Foreach participant, the assessment of PT, APTT, TT, IL-6, and IL-18, as well as ADMA and NO levels in serum, was performed four times—blood samples were collected immediately before the first IVA injection (control measurement), during and after the loading phase (2–3 days after the first injection, and immediately before and 2–3 days after the third injection) (Figure 1). Reference values for blood coagulation parameters of 13–17 s for PT, 24–36 s for APTT, and 14–20 s for TT have been accepted. Aflibercept was administered in the operating room in accordance with the standard protocol for intravitreal injections. Baseline visual acuity (BVA) was measured just before the first injection, while final visual acuity (FVA) was measured two months after the third injection.

### 2.3. Sample Collection

Blood samples were collected from the median cubital vein in sterile 2.7 mL tubes containing sodium citrate for TT, APTT, and PT assays, and 6 mL biochemical tubes for IL-6, IL-18, ADMA, and NO determinations. All blood samples within 30 min of the collection were centrifuged at 2000–3000 rpm for 20 min. TT, PT, and APTT determinations were made in an automatic coagulation analyzer. The required amounts of serum necessary to determine the parameters of the coagulation system have been used: APTT 50 µL; PT 100 µL; TT 100 µL. For the IL-6, IL-18, ADMA, and NO levels in the serum, the supernatant was poured into the four Eppendorf labeled tubes and then frozen at −80 °C until the enzyme-linked immunosorbent assay (ELISA) was performed for IL-6 (DRG International, Inc., Springfield, NJ, USA), IL-18 (Shanghai Sunred Biological Technology Co., Ltd., Shanghai, China), ADMA (Shanghai Sunred Biological Technology Co., Ltd., Shanghai, China), and NO (Shanghai Sunred Biological Technology Co., Ltd., Shanghai, China).

### 2.4. Statistical Analysis

The statistical analysis of the results obtained was carried out using the Statistica v.7.1 software (StatSoft Inc., Tulsa, OK, USA). The Shapiro Wilk test showed normal distribution of the variables. The differences between the variables were analyzed with the use of one-way analysis of variance (ANOVA) and further with posthoc analysis by Tukey’s HSD test. Pearson’s correlation analysis was performed to determine the relationship between the age of the enrolled patients and the initial and final serum concentrations of the measured parameters. The results of the study were presented as mean value +/− standard error (SE) values; with the *p*-value < 0.05 being considered statistically significant.

## 3. Results

### 3.1. Blood Coagulation Parameters

**Figure 2:**
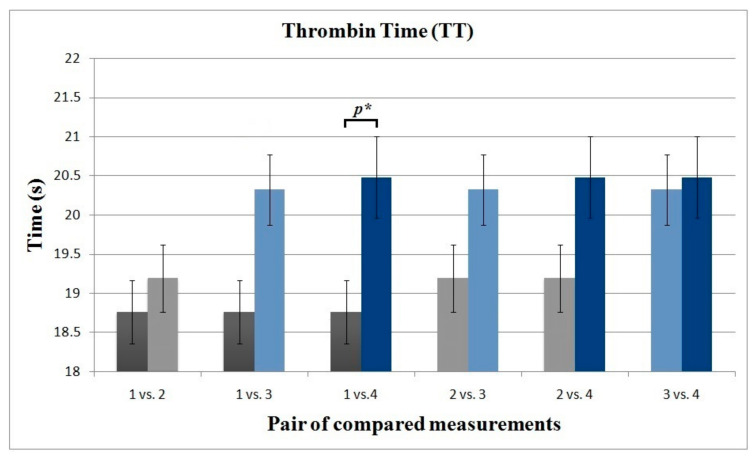
Column chart showing pairs of compared measurements for thrombin time (TT). p* statistically significant value (*p* = 0.041).

The initial measurements of the coagulation parameters examined, including PT, APTT, and TT, were within the normal range in all individuals (Table 2). A statistically significant increase was observed for TT in comparison to measurements obtained before the first and after the third IVA administration (*p* = 0.041) (Figure 2). No significant differences among other measures of TT were found. Similarly, there were no significant differences between the compared pairs of measurement points for PT and APTT; however, we observed a possible trend toward significance for APTT between the initial and final measurement (*p* = 0.053). Importantly, during the observation period, the average value of PT and APTT did not exceed the limit of the normal range at any of the time points; however, for TT the mean value both before and after the third administration slightly exceeded the standard reference range (20.32 ± 0.45 and 20.48 ± 0.52, respectively). The analysis of the correlation between the age of the patients and the values of PT, APTT, and TT showed no significant relationship for the initial (r = −0.351, *p* = 0.109; r = 0.052, 0.818; r = −0.294, 0.184, respectively) or the final (r = −0.081, *p* = 0.720; r = 0.136, 0.546; r = −0.055, 0.808, respectively) measurements.

### 3.2. AMDA and NO

There were no statistically significant differences in serum ADMA and NO levels between the measurement points of our study. The value of the correlation coefficient also did not show a significant relationship between age and ADMA and NO concentrations, before (r = −0.203, *p* = 0.365 for ADMA and r = 0.105, *p* = 0.642 for NO) or after (r = 0.061, *p* = 0.787 for ADMA and r = −0.009, *p* = 0.968 for NO) the initial loading phase of intravitreal aflibercept. At baseline, serum NO and ADMA values correlated positively; however, there was no statistical significance (r = 0.277, *p*-value = 0.212).

### 3.3. Interleukin 6 and Interleukin 18

**Figure 3:**
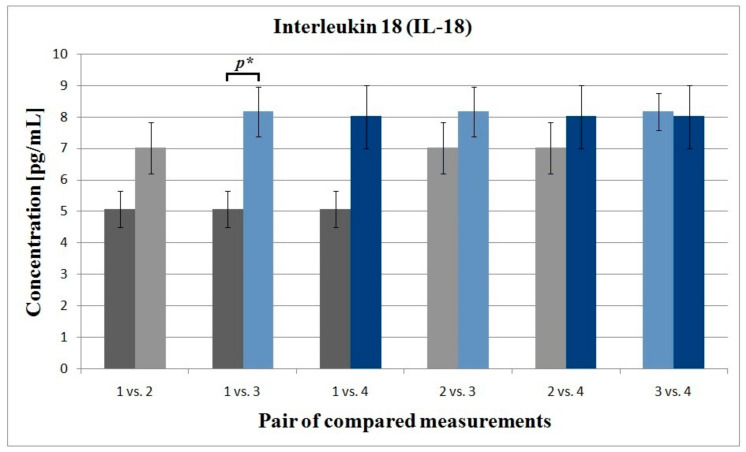
Column chart showing pairs of compared measurements for interleukin 18 (IL-18). p* statistically significant value (*p* = 0.037).

For serum IL-6 levels, no statistically significant differences between the measurement points were observed and the lowest *p*-value was demonstrated for comparison of measurements 2–3 days after the first and 2–3 days after the third IVA administration (*p* = 0.074). In contrast, for IL-18 a statistically significant difference between the measurements collected at the baseline and immediately before the third IVA administration was found (*p* = 0.037) (Figure 3). Furthermore, a result at the edge of significance for the initial measurement and measurement collected after the initial loading phase of IVA was also noted (*p* = 0.052). Similarly, there were no significant correlations between the age of the patients and the initial and final serum concentrations of IL-6 (r = −0.334, *p* = 0.129 and r = 0.042, *p* = 0.853, respectively) and IL−18 (r = 0.034, *p* = 0.881 and r = −0.342, *p* = 0.119, respectively). At baseline, a negative correlation between serum levels of IL-6 and IL-18 was demonstrated; however, it did not reach statistical significance (r = −0.022; *p*-value = 0.919). Similarly, there was also no significant correlation between the change in serum IL-18 concentration and improvement in the visual acuity (r = 0.268; *p*-value = 0.229).

All results of our study are summarized in Table 2. During the experiment, no side effects (e.g., increase in intraocular pressure, endophthalmitis, retinal detachment) or systemic (e.g., stroke, myocardial infarction, death) were reported in any of the participants.

## 4. Discussion

Aflibercept is a soluble, recombinant fusion protein consisting of human extracellular fragments of the second domain of the first VEGF receptor (VEGFR-1), the third domain of the second VEGF receptor (VEGFR-2), and the human IgG1-Fc domain [2]. Due to its specific composition aflibercept can inhibit both the VEGF-A isoform and in opposition to bevacizumab, ranibizumab, and brolucizumab, also VEGF-B, as well asplacental growth factor 1 (PlGF-1) and placental growth factor 2 (PlGF-2)—two factors that can act synergistically with VEGF-A in initiating pathological vascular remodeling and angiogenesis in patients with nAMD [2,3]. Aflibercept is also often referred to as the “VEGF trap molecule”, that term is associated with a higher affinity of aflibercept for VEGF and PlGF molecules compared to their native receptors—vascular endothelial growth factor receptor (VEGFR) and placental growth factor receptor (PlGFR), which results in the inhibition of their activation [2]. Animal studies revealed that after IVA administration, the vitreous half-life of aflibercept ranged from 2.2–4.58 days; while Stewart et al., based on the statistical model, estimated that the half-life can reach 7.13 days in human eyes [10]. The average serum half-life of aflibercept after intravenous administration of 2–4 mg/kg is about 5–6 days, compared to 20 days, 5.6 h, and 2 h for bevacizumab, brolucizumab, and ranibizumab, respectively [8,11]. It is postulated that the variation in serum half-life of anti-VEGF agents is caused bythe presence of the Fc domain in the aflibercept and bevacizumab structure, which has a protective function and prevents the degradation of these molecules by proteolytic enzymes of the endosomal pathway. It has been proved that, after intravitreal administration, anti-VEGF agents can pass into the systemic circulation and be detected in the non-injected eye, thyroid gland, heart, liver, femur bones, and kidney capillaries [8,12]. Systemic absorption may occur through the diffusion of drug molecules from the vitreous humor into the posterior chamber of the eye and subsequent absorption through the ciliary veins of the iris-ciliary body complex or through the outflow of the aqueous humor [13]. Another mechanism involves the active transport of the drug molecule across the blood-retinal barrier with the participation of Fc-receptors, which can bind molecules containing the IgG-Fc domain, allowing the anti-VEGF molecule to pass from the vitreous humor into the retina and further to the systemic circulation [8,13]. Avery et al. demonstrated that the three-monthly intravitreal injections of aflibercept at a dose of 2 mg and bevacizumab at a dose of 1.25 mg can lead to serum accumulation and achieve an IC50 value capable to inhibit VEGF activity in the systemic circulation. Studies in a rat model using positron emission tomography/computed tomography PET/CT to measure ocular and blood pharmacokinetics intravitreal ^89^Zr-aflibercept showed that the vitreous elimination half-life was 4.51 ± 0.72 h (0.19 +/− 0.03 days), while the blood elimination half-life was 3.18 ± 0.63 days with a maximum concentration of 25.40 Bq*µL^−1^ reached at 18.24 h (0.76 days) after injection, which is less than a quarter of the intravitreal dose (23.82%) [14]. These properties suggest that an early effect of IVA on peripheral blood system parameters observed two or three days after administration can be expected. Moreover, it has been also shown that among the available anti-VEGF agents, aflibercept possesses the highest ability to reduce the level of free VEGF in serum [8].

The main effect of the coagulation cascade is the conversion of soluble fibrinogen to insoluble fibrin and, as a result, the formation of a stable platelet-fibrin clot. Tests involving measurement of PT, APTT, and TT are routinely used to detect abnormalities in the external, internal, and common coagulation pathway, respectively [15]. APTT reflects the activity of the intrinsic coagulation pathway including factors such as high-molecular-weight kininogen (HMWK), prekallikrein (PK), factors VIII, IX, XI, and XII; PT is a measure of the efficiency of the extrinsic system, in particular the level of factor VII. Additionally, both APTT and PT may vary with changes in the concentration of common pathway factors including prothrombin, fibrinogen as well as factor V and factor X [16]. APTT and PT are the most commonly used screening tests for the assessment of the function of the coagulation system [14]. It is known that a reduction in PT and APTT values reflects a hypercoagulable state that is directly associated with an increased risk of thrombotic events [17,18]. In patients with APTT values below the normal range, elevated serum levels of the prothrombin fragment F1 + 2 have been observed. The F1 + 2 fragment is detached from prothrombin during its activation and conversion to thrombin—this process leads to the formation of thrombin, which allows further clot formation [19]. Importantly, a shortening of APTT has been shown to be associated with both an increased risk of thrombosis and rethrombosis in surviving patients [18]. It has been proven that a reduction in PT is associated with an increased serum prothrombin concentration, which may also promote hypercoagulability through increased thrombin production and facilitated clot formation [18]. Conversely, an abnormal increase in the value of these indicators may be a risk factor for pathological bleeding [15].

To date, four studies assessing the influence of intravitreal injections of anti-VEGF agents on the coagulation parameters have been published [9,13,20,21]. In patients with proliferative diabetic retinopathy (PDR), the effect of IVB on prothrombin time was evaluated [13], while another study evaluated clotting time, serum fibrinogen level, and blood viscosity in patients with CNV treated with IVR [9]. In the third study, changes in APTT and PT were measured in patients with nAMD who received IVA [20]. Georgakopoulos et al. assessed the effect of IVA on platelet count, PT, APTT, and fibrinogen, D-dimer, and protein C and S levels in the serum of patients with AMD [21]. Altinkaynak et al. revealed a decrease in APTT and PT values both one month after the first IVA injection and one month after the second injection in patients with nAMD; however, in both measurement points the results were not statistically significant [20]. The results of other study showed a significant reduction in APTT and an increase in the low, medium, and high serum shear rate measured after the first week of IVR injection in patients with CNV compared to the control group; this difference was not demonstrated a month after the first and or a month after the second IVR administration. It is worth emphasizing that despite the significant difference between the test group and the control group, APTT in both groups was within the normal range [9]. Qian et al. investigated the effect of IVB injections on prothrombin time in three groups of patients—the first group included patients with PDR, the second group included non-diabetics receiving IVB, and PDR was found in the control group (patients did not receive IVB). The results showed a reduction in prothrombin time in the PDR group within 2 weeks after IVB injection; however, the prothrombin time didnot exceed the normal limit in any of the measurements [13]. Georgakopoulos et al. showed no statistically significant changes in the value of any of the parameters tested, one week after and one month after IVA administration [21]. Similarly, our results showed no significant changes in PT or APTT in patients following IVA. Nevertheless, a suggestive upward trend compared to the APTT values before the first IVA administration and after the third injection was noted (*p* = 0.053). This phenomenon can probably be explained by the results of the study conducted by Yamashita et al., which showed that administration of 2 mg IVA in patients with nAMD may cause a significant decrease in both von Willebrand factor antigen (vWF:Ag) and VEGF-A levels, but no effect on the level of ADAMTS13 in the serum after the first week and persisting one month after IVA administration [22]. The Von Willebrand factor (vWF) is a multimeric glycoprotein synthesized by megakaryocytes and Weibel–Palade bodies located in endothelial cells [15], and its release is regulated by the concentration of VEGF in serum and can be inhibited in the case of VEGF deficiency [22]. The main function of vWF in the hemostasis process is the regulation of platelet adhesion to damaged vascular endothelium by binding on its surface specific receptors for platelet glycoproteins and collagen named glycoprotein Ib (GPIb) and glycoprotein IIB/IIIa(GPIIb/IIIa). vWF also has a protective function against factor VIII preventing its premature degradation in serum due to the stabilization of factor VIII molecular structure, inhibition of its proteolytic degradation, inhibition of factor VIII binding to active factor IX and prevention of factor VIII removal by scavenger receptors [23]. Hence, a disturbance inthe amount of vWF in the serum may lead to a decrease in the serum half-life of factor VIII and, consequently, lead to an abnormal course of the intrinsic coagulation pathway manifested by prolongation of APTT. Additionally, APTT and PT values were not increased before the first IVA injection. It is worth emphasizing that the values of both discussed parameters did not exceed the normal range at any of the measurement points of our study.

The main test used to assess the common coagulation pathway is thrombin time (TT), which is defined as the clotting time of citrate plasma after the addition of thrombin—a factor necessary for the conversion of fibrin to fibrinogen. In clinical practice, the assessment of TT is used to assess fibrinogen deficiency, impairment of its activity, and to detect the presence of thrombin inhibitors such as heparin, direct thrombin inhibitors (e.g., dabigatran), and anti-thrombin antibodies or thrombin-induced antibodies in the serum—these factors cause a prolongation of TT [15]; while a reduction in TT may reflect astate of hyperfibrinogenemia that is associated with an increased cardiovascular risk as well as a greater possibility of myocardial infarction and death, as shown by the results of the Framingham Study [24,25]. Furthermore, elevated plasma fibrinogen can lead directly to both venous and arterial thrombosis and lead to thrombolytic resistance by promoting increased fibrin formation and clot stability [24].

Our results showed a statistically significant difference in thrombin time compared to the baseline measurement and the measurement after the third IVA administration (*p* = 0.041). It can be assumed that the prolongation of TT may result from a decrease in serum fibrinogen (hypofibrinogenemia) or its structural abnormalities resulting in an abnormal function (dysfibrinogenemia) [15]. Georgakopoulos et al. revealed no significant changes in serum fibrinogen concentration at 7 and 30 days after a single IVA injection, and, thus, the influence of IVA on fibrinogen function should be considered [21]. Fibrinogen is a glycoprotein synthesized in the liver consisting of three heterodimers (αA, βB, γ) connected by disulfide bridges and has the ability to bind to the GPIIb/IIIa receptor located on the surface of platelets and megakaryocytes [26]. Previous studies have shown that aflibercept as a molecule containing the Fc domain of human IgG1 has the ability to interact with the GPIIb/IIIa receptor causing its upregulation by affecting the integrin signaling pathway mediated by FcyRIIa (CD32) [6,27]; however, other interactions between aflibercept and platelets receptors cannot be excluded and further studies are necessary to fully understand the impact of the Fc domain in this issue, and, particularly, to investigate the possible effects on fibrinogen functions and the coagulation process. To the best of our knowledge, this study is the first to evaluate the effect of intravitreal injection of the anti-VEGF agent on the thrombin time.

Nitric oxide (NO) is a small signaling molecule that plays a key role in regulating the function, maintaining tension, and the structure of the endothelium of blood vessels. In addition to the vasodilator function, NO inhibits platelet adhesion and aggregation, as well as the adhesion of monocytes and leukocytes, and can reduce both the production of oxygen free radicals and the oxidation of low-density lipoprotein (LDL)-cholesterol [28]. In mammals, the nitric oxide synthase (NOS) enzyme plays a key role in NO synthesis and occurs in three isoforms: endothelial (eNOS)—which plays a key role in the maintenance of cardiovascular homeostasis—as well neuronal (nNOS), and inducible (iNOS) [5,29]. In the cardiovascular system, NO is synthesized and released by vascular endothelial cells, vascular smooth muscle cells, endocardium, and also by cardiac myocytes and platelets [29]. Besides vasodilation, the main function of endothelial-derived NO is to prevent platelet adhesion to the vascular walls; platelet-derived NO inhibits the recruitment of platelets and platelet-platelet aggregation in the forming clot—both of these properties prevent vascular thrombosis [30].

ADMA has the ability to inhibit eNOS activity leading to decreased NO bioavailability for endothelial cells and can cause vasoconstriction. Moreover, ADMA presents proatherogenic properties, which may increase cardiovascular risk, but also contribute to the development of AMD. On the other hand, the results of current studies have shown that ADMA can inhibit VEGF-induced angiogenesis [31], which may prevent the development of nAMD. The relationship between ADMA levels in serum and the occurrence of AMD remains unclear. Keles et al. showed that patients with nAMD are characterized by a higher concentration of ADMA, as well as reduced eNOS activity and a lower serum concentration of NO in comparison to individuals without retinal disorders [32]. Other studies also showed an increase in the level of ADMA in the serum of patients with AMD [33]; while Pinna et al. did not reveal a significant difference in AMDA serum levels in early or in nAMD compared to healthy individuals [34]. 

The role of NO in the pathogenesis of AMD is also not fully understood. Previous studies showed inconsistent results—both increased and decreased, as well as no change in serum NO levels in AMD patients were observed [35]. Animal studies have shown that systemic administration of PTK787/ZK222584—a blocker of VEGF-R—leads to a decrease in eNOS activity and a decrease in the bioavailability of NO [36]. Conversely, the research conducted by Sümbül et al. did not show a reduction in serum NO after systemic bevacizumab treatment in patients with metastatic colorectal cancer [37]. We demonstrated that before IVA, both ADMA and NO serum levels in nAMD patients were within the populational norms [38,39]. Our study is the first to evaluate the effects of intravitreal anti-VEGF on levels of ADMA and NO in serum and we did not observe any significant changes in serum AMDA or NO levels.

Interleukins, small protein molecules, belong to the cytokine family and regulate cell signaling processes during inflammatory response in the body [35]. Both IL-6 and IL-18 belong to the group of proinflammatory cytokines and are characterized by the ability to stimulate abnormal angiogenesis; however, animal studies have shown that IL-18 may be a protective factor against the development of CNV [40,41,42]. It was shown that serum levels of IL-6 may correlate with the occurrence and progression of AMD [35]. Additionally, animal studies using the microbead occlusion model of ocular hypertension have shown that IL-6 plays a significant role in RGC axon degeneration and in IOP-dependent visual acuity deterioration [43]. Interestingly, an IL-6 inhibitor, tocilizumab may be a promising therapeutic option in patients with refractory pseudophakic CME, leading to a significant improvement in the structural parameters of the fovea and visual acuity [44]. On the other hand, studies in a rodent model of retinal detachment have demonstrated the neuroprotective properties of IL-6 against photoreceptors [45]. Animal studies have shown that IL-18 has the ability to counteract vascular leakage, reduce vascular permeability, and reduce the severity of VEGF-induced retinal and subretinal neovascularization. In addition, IL-18 improves the tightness of the blood-retinal barrier (BRB) junctions by increasing the expression of their specific protein—claudin 5, and also enhances the expression of thrombospondin 1—a protein with anti-angiogenic properties [42].

Previous studies have shown that elevated serum levels of both IL-6 [46,47,48] and IL-18 [49,50,51] are associated with a higher cardiovascular risk in patients of different risk groups. In both human and animal studies, an association between the occurrence of deep vein thrombosis (DVT) and increased expression of IL-6 has been demonstrated [52]. IL-6 induces the dysfunction of the vascular endothelium by increasing the production of molecules involved in the regulation of inflammatory processes on its surface and increasing its permeability (e.g., ICAM-1, VCAM-1, CCL-2, CCL-3). In addition, IL-6 enhances the synthesis of molecules with vasoconstriction properties (e.g., AT II, ET-1) [52,53]. The influence of these molecules can cause endothelial dysfunction and lead to thrombus formation. Additionally, IL-6 may induce an increase in the expression of fibrinogen and factor VIII [54], as well as thrombogenesis and platelet hyperactivity, which may promote both arterial and venous thrombosis [55].

In vitro studies with the use of rat inferior vena cava model of DVT and human umbilical vein endothelial cells (HUVECs) culture showed an association between increased IL-18 expression and DVT occurrence through an increase in vWF and *p*-selectin expression, as well as a decrease in tissue plasminogen activator (tPA) [56]. A study by Trø seid et al. showed that IL-18 may be an independent marker of the risk of cardiovascular disease in the elderly population with metabolic syndrome by mediating a synergistic pro-inflammatory and hyperglycemic effect [57].

Our results showed that at the baseline, both IL-6 and IL-18 serum levels were within the populational norms [58]. There were no significant changes in the IL-6 serum levels after IVA administration; while for IL-18, a statistically significant increase between measurements at the baseline and immediately before the third IVA administration has been observed. Sato et al. demonstrated a significant increase in IL-6 levels in the aqueous humor of nAMD patients after three monthly IVA administrations [4]; while other studies have shown a reduction in IL-6 levels in patients with central retinal vein occlusion (CRVO) after IVA and IVR administration, as well as after two injections of IVR in patients with nAMD [59,60]. On the other hand, higher concentrations of IL-18 in the aqueous humor of patients with macular edema secondary to CRVO treated with ranibizumab have been shown. Interestingly, these changes correlated with better final visual improvement [42]. Similarly, in our study group, a significant improvement in visual acuity was also observed (Table 1); however, statistical analysis only showed a weak positive correlation between these two variables. Despite this, our results have shown that the effect of the increase in IL-18 serum concentration in individuals undergoing treatment with anti-VEGF agents should be investigated. Further research is needed in order to explain whether it is a clinically significant association, and verify the potential molecular mechanisms underlying this phenomenon.

Our study also has three main limitations. Firstly, the size of the study sample is relatively small, which is mainly a result of the strict exclusion criteria for participants. However, we decided to use such stringent conditions to minimize the potential influence of other factors on the obtained results. Secondly, the possible influence of external factors, such as the potential effect of the initial nutritional status as well as the patients’ diet and lifestyle during the experiment period on the measured concentrations of ADMA, NO, IL-6, and IL-18 in the peripheral blood should be considered [61,62,63]. Additionally, in our study, one of the exclusion criteria was uncontrolled hypertension; however, there are reports of changes in ADMA and NO levels in patients with well-controlled arterial hypertension [64]. Similarly, in elderly patients, a more frequent occurrence of osteoarthritis is observed, the presence of which may also influence the concentrations of endothelial function parameters and pro-inflammatory cytokines in peripheral blood [65,66]. Moreover, hormonal disturbances, which often occur in the older population, especially abnormal levels of estrogen and testosterone, may affect the blood coagulation system [67]. Finally, although the presence of inflammatory symptoms was ruled out on the basis of the medical history before each IVA injection, the determination of CRP at each of the study time points could also be performed as an additional test to exclude the potential influence of inflammatory processes on the results obtained. Apart from the aforementioned limitations, there are no studies clearly confirming or excluding the hypothesis that intravitreal injection itself may lead to an increase in the concentration of pro-inflammatory cytokines in the peripheral blood as a result of local tissue trauma.

## 5. Conclusions

In conclusion, we found a statistically significant difference showing the increase in TT values between measurements before the first IVA injection and after the initial loading phase. The results of our study suggest that IVA administration may have an effect on the common blood coagulation pathway, which manifests as a prolongation of the TT value. Moreover, we have shown a significant increase in serum concentration of pro-inflammatory cytokine—IL-18 during the initial loading phase of IVA, but we did not observe significant changes in the serum concentration of another pro-inflammatory cytokine—IL-6. Further research is necessary to clarify whether the observed effect of IVA administration on the TT value and systemic level of IL-18 is related to the primary or indirect effect of the aflibercept molecule.

## Figures and Tables

**Table 1:** Clinical and demographic characteristics of individuals included in the study.

Number of Patients (Eyes), N (%)	
All	22
Female	13 (59.01)
Male	9 (40.9)
Age in years (mean value)	
Female	78.69
Male	76.89
Range	66–99
Visual Acuity (mean LogMAR)	
BVA ^a^	0.2944
FVA ^b^	0.2399
*p*-value ^c^	0.001

**Abbreviations:** BVA—baseline visual acuity; FVA—final visual acuity; ^a^—measured with the use of Snellen eye test charts just before first IVA injection; ^b^—measured with the use of Snellen eye test charts two months after the third injection of IVA; ^c^—comparison of the baseline visual acuity and final visual acuity of tested patients.

**Table 2:** Comparison of results of blood coagulation parameters, IL-6, IL-18, ADMA, and NO serum levels measured atdifferent time points.

Compared Measurements	Parameter																				
	PT			APTT			TT			IL-6			IL-18			ADMA			NO		
	MV (s)	SE	*p*-Val.	MV (s)	SE	*p*-Val.	MV (s)	SE	*p*-Val.	MV (pg/mL)	SE	*p*-Val.	MV (pg/mL)	SE	*p*-Val.	MV (µmol/L)	SE	*p*-Val.	MV (µmol/L)	SE	*p*-Val.
1 2	14.87 14.79	0.22 0.23	0.981	26.31 27.83	0.35 0.60	0.311	18.76 19.19	0.40 0.43	0.891	11.74 11.13	1.54 1.17	0.278	5.06 7.02	0.58 0.81	0.317	0.87 0.79	0.10 0.12	0.946	9.38 6.86	1.23 0.83	0.387
1 3	14.87 15.20	0.22 0.20	0.864	26.31 27.75	0.35 0.65	0.360	18.76 20.32	0.40 0.45	0.076	11.74 11.54	1.54 1.82	0.702	5.06 8.16	0.58 1.00	**0.037**	0.87 0.76	0.10 0.06	0.874	9.38 10.01	1.23 1.12	0.979
1 4	14.87 15.40	0.22 0.45	0.584	26.31 28.58	0.35 0.78	0.053	18.76 20.48	0.40 0.52	**0.041**	11.74 12.21	1.54 2.58	0.385	5.06 8.01	0.58 0.78	0.052	0.87 0.91	0.10 0.13	0.992	9.38 9.73	1.23 1.24	0.996
2 3	14.79 15.20	0.23 0.20	0.758	27.83 27.75	0.60 0.65	0.891	19.19 20.32	0.43 0.45	0.291	11.13 11.54	1.17 1.82	0.330	7.02 8.16	0.81 1.00	0.719	0.79 0.76	0.12 0.06	0.997	6.86 10.01	0.83 1.12	0.199
2 4	14.79 15.40	0.23 0.45	0.455	27.83 28.58	0.60 0.78	0.802	19.19 20.48	0.43 0.52	0.186	11.13 12.21	1.17 2.58	0.074	7.02 8.01	0.81 0.78	0.792	0.79 0.91	0.12 0.13	0.877	6.86 9.73	0.83 1.24	0.272
3 4	15.20 15.40	0.20 0.45	0.961	27.75 28.58	0.65 0.78	0.750	20.32 20.48	0.45 0.52	0.902	11.54 12.21	1.82 2.58	0.459	8.16 8.01	0.81 0.78	0.899	0.76 0.91	0.06 0.13	0.774	10.01 9.73	1.12 1.24	0.998

Measurement: 1—immediately before the first IVA injection; 2—2–3 days after the first IVA injection; 3—immediately before the third IVA injection; 4—2–3 days after the third IVA injection; In bold—statistically significant *p*-value (*p* < 0.05); Abbreviations: MV, mean value; SD, standard deviation; *p*-val., *p*-value; PT, prothrombin time; APTT, activated partial thromboplastin time; TT, thrombin time; IL-6, interleukin 6; IL-18, interleukin 18; ADMA, asymmetric dimethylarginine; NO, nitric oxide.

## Data Availability

Detailed data supporting the results can be provided by the corresponding author upon reasonable request.
